# Effects of 3S business intelligence systems for nursing students: a repeated-measures randomized control trial

**DOI:** 10.1186/s12912-023-01686-y

**Published:** 2024-01-11

**Authors:** Ji-Young Lim, Seulki Kim, Juhang Kim, Seonhee Kim

**Affiliations:** 1 Department of Nursing, Inha University, 100 Inha-Ro, Michuhol-Gu, Incheon, 22212 South Korea; 2 Yeonsuhanmaeum Day Care Center, Biryu-Daero, Yeonsu-Gu, 202 Incheon, South Korea; 3 Department of Nursing, Seojeong University, 27 Seojeong-Ro, Eunhyeon-Myeon, Yangju-Si, Gyeonggi-Do, South Korea; 4 Gangseosmile Nursing Home Care Center, 2F 45, Gonghang-Daero 59-Gil, Gangseo-Gu, Seoul, South Korea

**Keywords:** Student, Nursing, Internet-based intervention, Entrepreneurship, Simulation

## Abstract

**Background:**

The growing need for healthcare services as a result of a consistently rising prevalence of chronic diseases and rapid population aging calls for a new set of activities and practices. Therefore, we developed a program—3S (Simple, Smart, and Speed) Business Intelligence Systems (3S-BIS), which is an ERP software system that helps nursing business to support nursing entrepreneurship —and analyzed its effects on nursing students.

**Methods:**

A repeated-measures randomized controlled trial was performed with two groups: experimental (*n* = 29) and control (*n* = 30) groups. The former group underwent the five-day 3S-BIS education program. Each session comprised four components: lectures 1 and 2, simulation case study, and debriefing. Post-tests were performed immediately post-intervention and four and eight weeks later. The effectiveness was measured using the following variables: simulation design assessment, evaluation of educational practices in simulation, education satisfaction, self-efficacy for learning, and entrepreneurship. The differences before and after intervention between the experimental and control groups were analyzed using the Friedman test. The Mann–Whitney U test was used for comparisons between groups at each time point, and the Wilcoxon signed-rank test was used for comparisons within groups at each time point.

**Results:**

Post-intervention (8 weeks after intervention), the experimental group demonstrated higher simulation design assessment (z = -3.88,* p* =  < .001), evaluation of educational practices in simulation (z = -3.34, *p* = .001), education satisfaction (z = -3.11,* p* = .002), self-efficacy for learning (z = -3.04,* p* = .002), and entrepreneurship (z = -2.15,* p* = .031) compared to controls. Furthermore, simulation design assessment score in the experimental group significantly differed between T1 (immediately after intervention) and T0 (baseline), and between T3 (8 weeks after intervention) and T0. Evaluation of educational practices in the simulation, education satisfaction, and self-efficacy also significantly differed between T1 and T0, and between T3 and T0. Entrepreneurship significantly differed between T3 and T2 (4 weeks after intervention), and between T3 and T0.

**Conclusions:**

The 3S-BIS program contributes to enhancing nursing start-up competency. Subsequent studies should evaluate the effects of the program on nurses who work in home healthcare services.

## Background

At the turn of the fourth Industrial Revolution, start-ups have emerged as a new growth engine for countries [1]. University curricula have also been changing to adapt to these changes, and entrepreneurship education has been actively spreading. The South Korean government has been promoting start-ups as a national policy project and has actively implemented relevant policies [2]. Moreover, in South Korea, the need for entrepreneurship education that can support decision-making, such as creation and innovation processes that reflect the ecosystem of each industry, entrepreneurship, problem-solving skills, and search for business opportunities, has been emphasized.

The healthcare industry is a rapidly growing market with a global popularity of start-ups in healthcare products [3], healthcare-related artificial intelligence businesses [4], and nursing services [5]. In South Korea, long-term care (LTC) insurance for the elderly was implemented in July 2008, and it laid the legal grounds for when nurses can establish an independent LTC facility [6]. The growing need for healthcare services as a result of a consistently rising prevalence of chronic diseases and rapid population aging [7] calls for a new set of activities and practices [8]. Nurses play key roles in preventive primary healthcare, and the importance of entrepreneurial competence will only increase among nurses [9].

According to Vannucci and Weinstein, the challenges that nursing professionals face in starting their business are primarily those pertinent to their inadequate managerial competencies, such as budget, finance accounting, and marketing [5]. Further, experiences in nursing start-up businesses, strong networking, and business-related education have been emphasized for nursing entrepreneurs to adapt to their changed roles [10]. For nursing entrepreneurship, the entrepreneurship education during undergraduate years has been extremely effective [10]. Newman et al. [11] and Salminen et al. [12] posit that entrepreneurship education should reflect the target population’s characteristics and use various teaching concepts and methods. Noh et al. [13] emphasized that both theoretical and practical education, such as the basics of entrepreneurship management and management strategy, were necessary subjects for nursing students to learn in entrepreneurship education.

Businesses must adapt quickly to survive amid rapidly evolving business environment in recent years. To this end, real-time processing and prompt decision-making are essential, for which an Enterprise Resource Planning (ERP) system and a business intelligence (BI) system are used [14]. Hence, it is important to ameliorate the management of home healthcare businesses by developing management support programs tailored to home health businesses, which may ultimately bridge the gap between the nursing profession and start-ups. To this end, BI software tailored to the features and environment of the home health industry is needed.

Consequently, the importance of entrepreneurship education is being emphasized from the undergraduate level so that nursing students can act as nursing entrepreneurs in the field of community nursing. However, there is still a lack of nursing entrepreneurship education curriculum or educational media based on BI that can support entrepreneurship education. Thus, this study develops and evaluates the effectiveness of a 3S (Simple, Smart, and Speed)-BI System (3S-BIS) among nursing students to encourage and prepare community-based home care nursing start-ups.

### Aims

This study is the first of two intervention studies that evaluates the effects of a 3S-BIS developed with funding from the National Research Foundation of Korea. The two intervention studies include a randomized controlled trial (RCT) with repeated measures to investigate the effects of the 3S-BSI program on enhancing entrepreneurial competency and retaining of the effects in nursing students, and a study that investigates the effects of the 3S-BIS program on enhancing entrepreneurial competency of nurses preparing to start a nursing business and the differences in effects according to the educational medium. This study investigates the effects of the 3S-BIS program on simulation design assessment, evaluation of educational practices in simulation, learners’ satisfaction, self-efficacy for learning, and entrepreneurship in nursing students.

### Study hypotheses

Against the background discussed above, the following hypotheses were proposed:

- Hypothesis 1. The experimental group that participates in the 3S-BIS program will have higher simulation design assessment (1–1), evaluation of educational practices in simulation (1–2), education satisfaction (1–3), self-efficacy for learning (1–4), and entrepreneurship (1–5) than the control group.
- Hypothesis 2. Simulation design assessment (1–1), evaluation of educational practices in simulation (1–2), education satisfaction (1–3), self-efficacy for learning (1–4), and entrepreneurship (1–5) of the experimental group that participates in the 3S-BIS program will differ across time points.

## Methods

### Study design

This study used a repeated-measures RCT design to evaluate the effects of a 3S-BIS program among nursing students.

### Participants

Nursing students from 16 schools nationwide were enrolled. To recruit participating students, we sought cooperation from nursing departments located in large cities and used recruitment notices on bulletin boards within the departments. The inclusion criteria included third- and fourth-year nursing students who were interested in starting a nursing business from the perspective of their future career. The exclusion criteria included the first- and second-year students who were still in the process of exploring their future career paths.

Sample size was determined using G*Power 3.1. Power of 0.80, significance level of 0.05, an effect size of 0.25 [15], and three repeated measures were set for a two-group repeated-measures analysis of variance. Furthermore, power of 0.80, significance level of 0.05, and an effect size of 0.25 were set for analysis of within-between interactions and differences between the two groups. The sample size required for the former analysis was 36 (18 for each group) and that required for the latter analysis was 50 (25 for each group). A dropout rate of 20% was anticipated; thus, we aimed to recruit 60 participants (30 in each group).

A research assistant not related to the participants assigned a number to each of the 59 eligible participants. Participants were assigned to each of the two groups in a 1:1 ratio by setting the allocation ratio at 50% through random selection using SPSS version 26.0 (IBM Corp., Armonk, NY, USA). After assigning participants to two groups, a coin was tossed, with the heads as experimental group and tails as the control group. The group allocation results were notified to participants individually via a text message or email. Blinding of the researchers and participants was impossible. To reduce the risk of bias, a research assistant, who was not involved in the study, coded the online questionnaire data for the experimental and control groups, input data on Microsoft Excel, and deleted the data column pertinent to the experimental group. Thus, the data analyzer was blinded from the group allocation.

**Fig. 1:**
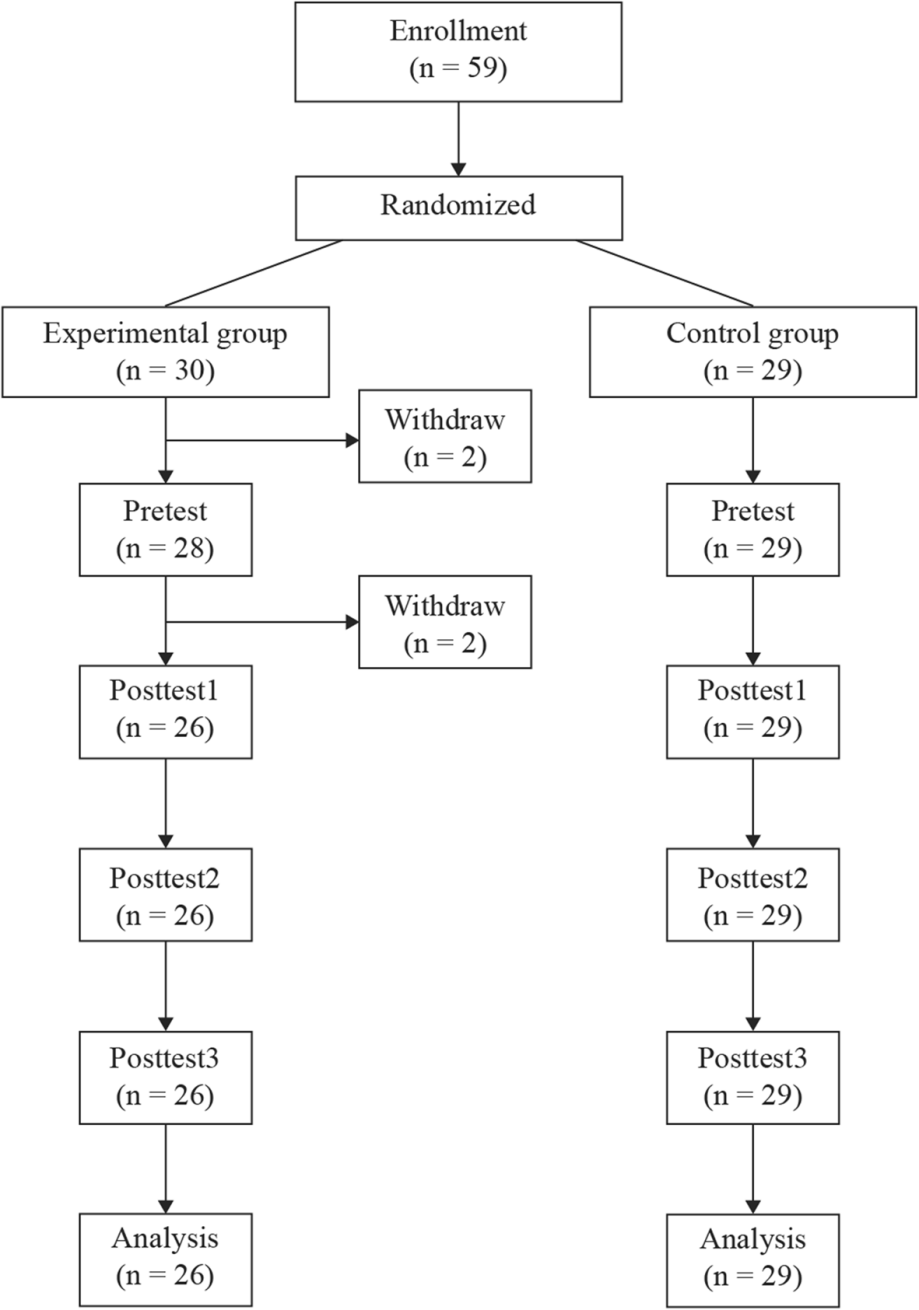
Flowchart of post-recruitment study participation by the experimental and control groups

Thirty participants in the experimental group and 29 in the control group provided written informed consent to participate. During the intervention, two participants from the experimental group withdrew prior to the pre-test owing to the overlap of schedule with clinical practicum. After the pre-test, two more participants from the experimental group withdrew from the study as the program content did not meet their expectations. Consequently, 26 participants in the experimental group and 29 in the control group completed the 3S-BIS program and three post-test rounds (Fig. 1).

### Measurements

#### Simulation design assessment

The simulation design was assessed using the instrument developed by the NLN/Laerdal simulation research team [16]. The instrument comprises 15 items in five domains (objectives/information, support, problem-solving, feedback, and fidelity). Each item is rated on a five-point Likert scale, and a higher score indicates more positive evaluation of the simulation design. The Cronbach's α was 0.92 at the time of development, 0.97 in a study on learning flow that used the Korean version of the instrument after removing the “support” domain [17], and 0.97 in this study.

#### Evaluation of educational practices in simulation

Educational practices in simulation were evaluated using the instrument developed by NLN/Laerdal [16]. This instrument comprises 16 items in four domains (active learning, collaboration, diverse ways of learning, and high expectations). Each item is rated on a five-point Likert scale, and a higher score indicates more positive evaluation of the educational practices in the simulation. The Cronbach's α was 0.86 at the time of development [16], 0.97 in a study that used the Korean version of the instrument [17], and 0.97 in this study.

#### Education satisfaction

Education satisfaction was assessed using an eight-item scale developed by Park [18] that uses a five-point Likert scale. The Cronbach's α was 0.95 at the time of development and 0.94 in this study.

#### Self-efficacy for learning

Self-efficacy for learning was assessed using the instrument developed by Ayres [19] and modified and adapted by Park and Kwon to assess academic self-efficacy in nursing students [20]. This ten-item scale uses a seven-point Likert scale. The Cronbach's α was 0.94 at the time of development [19], 0.93 in the study that used the Korean version of the instrument [20], and 0.97 in this study.

#### Entrepreneurship

Entrepreneurship was assessed using the instrument developed by Yoo et al. [21] and modified and adapted by our research team according to our study population and study objectives. This 12-item instrument uses a five-point Likert scale, and a higher score indicates greater entrepreneurship. The Cronbach’s α was 0.81 in a previous study [9] and 0.88 in this study.

### Composition of 3S-BIS

#### Contents

The 3S-BIS is an ERP software system that helps home health businesses to improve their management by integrating human resources management; inventory management; and operations management based on a cost-volume-profit analysis, a key factor in management performance. The software also assists in data management, analysis, and integration in accordance with a specific objective, such as revenue, cost, and profit management. The 3S in the 3S-BIS refers to (1) Smart: a functional aspect, where fragmented data are integrated to automatically produce information needed to achieve the business objectives; (2) Speed: a process aspect, where data obtained on site can be immediately sent via a smartphone app or results of an analysis are available for view using a smartphone app, in consideration of the fact that most activities of home health businesses are primarily performed outside the office; (3) Simple: a user aspect, where the software is user-friendly, involving automatic calculation with minimally required manual data entry.

#### Design

The 3S-BIS program was designed in three phases. In the development phase, a prototype was developed, and expert content validity evaluation was performed to develop a model most appropriate for the nature of home health businesses. Furthermore, considering that most activities of home health businesses are performed outside the office, a cloud service and web-based application that allows easy access to the server anywhere and at any time were developed. In the verification phase, one-years’ worth of management and operation data of one home healthcare business was uploaded for a test run to verify the feasibility of the software. Any errors, barriers, and other problems were evaluated and rectified to finalize the 3S-BIS software.

The 3S-BIS comprises five modules: Business operation, employee management, client management, inventory status, and operation performance. 1) In the business operation module, the overall operational status, cost of labor, operational and management costs, and material cost are entered and can be viewed, and business revenue and cost are visualized as graphs and tables. 2) The employee management module shows the overall work performance and work schedules of employees. Each employee’s performance, personal plans, and goal achievement plans are shown. 3) The client management module shows the status by LTC insurance grade as well as client’s individual schedule and treatment histories. 4) The inventory status module shows the current quantity of supplies and consumables and whether the current inventory is adequate. 5) The summary category of the operation performance module provides a monthly summary of the entered data as a pie chart, and users can simulate their target revenue achievements by entering various changes from the current business performance (Table 1).

**Table 1:**
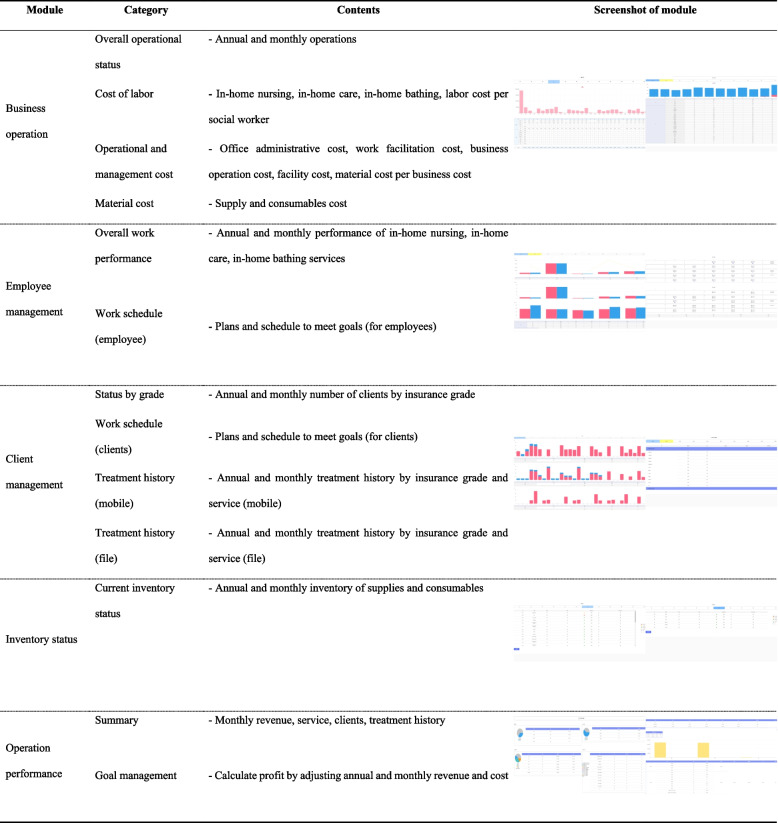
3S-BIS Blueprint

### Intervention

A web domain for 3S-BIS was launched (https://3s-bis-dev.thingsw.com). The intervention consisted of 100-min education provided daily for five days. Each education session consisted of four components: lecture 1 and 2, simulation case study, and debriefing. Lecture 1 was a 30-min lecture about the foundations of management, and lecture 2 was a 20-min lecture about the features and utilization of the 3S-BIS software in relation to the contents of lecture 1. Simulation case study was a 30-min session in which the users can add or modify existing data on 3S-BIS to simulate the changes in the business. A video conference was held to allow participants to present their results, and the correct answers were shared along with feedback. After the session, participants shared any thoughts about the software and implications, and a 20-min debriefing with a question-and-answer session was held. The control group was given an ID and password that granted them access to the 3S-BIS website, such that they could access the software and learned about it for the same five-day intervention period.

### Data collection

Data were collected from July 12, 2022, to September 18, 2022. A research assistant delivered and collected the questionnaire via email or social media, and the data were collected from the Department of Nursing in 16 schools. After the pre-test, the experimental group underwent the 3S-BIS education and practice for five days. A post-test was performed immediately after the 3S-BIS intervention and four and eight weeks after the intervention [15].

### Data analysis

The data obtained were analyzed using the IBM SPSS/WIN 26.0 software as follows:

1. Participants’ general characteristics were presented as real number and percentage, and homogeneity was tested using the chi-square and Mann–Whitney U tests.
2. Both groups’ simulation design assessment, evaluation of educational practices in simulation, education satisfaction, self-efficacy for learning, and baseline homogeneity in entrepreneurship were analyzed with the Mann–Whitney U test.
3. Differences in simulation design assessment, evaluation of educational practices in simulation, education satisfaction, self-efficacy for learning, and entrepreneurship before and after the intervention between the experimental and control groups were analyzed with the Friedman test, and intergroup differences at each time point were analyzed using the Mann–Whitney U test. Within-group comparisons at each time point were performed using the Wilcoxon signed-rank test. To reduce the risk of type 1 error, statistical significance was adjusted to 0.017 for Bonferroni correction [22].
4. The reliability of the instruments was tested using Cronbach’s alpha.

### Ethical considerations

This study was approved by the Institutional Review Board of Inha University (no. 220530-1A). Participants were recruited by posting a recruitment announcement on online communities or blogs of nursing schools. Volunteering participants were given a verbal explanation about the objectives of the study and data collection. After informing participants about the right to not participate in the study and freedom to withdraw from the study at any time, written informed consent was obtained from those who expressed willingness to participate in the study. All participants were given 25 USD as compensation. The control group was instructed about the 3S-BIS software and given an opportunity to use the software after the experimental group completed their questionnaires.

## Results

### General characteristics and baseline homogeneity

Both the experimental (*n* = 23, 88.5%) and control groups (*n* = 24, 82.8%) predominantly comprised of women, and the mean ages were 25.1 ± 8.1 years and 23.5 ± 2.9 years, respectively. The experimental group had 13 third-year and 13 fourth-year students (50%), while the control group had 15 (51.7%) third-year and 14 (48.3%) fourth-year students. Twenty-four (92.3%) participants in the experimental group had part-time work experience, while 28 (96.6%) in the control group had part-time work experience. The mean monthly allowance was 535.4 ± 245 thousand Korean won (KRW) in the experimental group and 568.3 ± 290.8 thousand KRW in the control group. There were participants without prior start-up education in the experimental (*n* = 23, 88.5%) and control groups (*n* = 26, 89.7%).

The general characteristics of the two groups were statistically homogeneous at the baseline. The study parameters, including simulation design assessment (z = 0.20, *p* = 0.839), evaluation of educational practices in simulation (z = 1.49, *p* = 0.137), (z = 0.19, *p* = 0.846), self-efficacy for learning (z = -0.46, *p* = 0.648), and entrepreneurship (z = -0.46, *p* = 0.642), were also statistically homogeneous at the baseline (Table 2).

**Table 2:** Demographic characteristics (*N* = 55)

Characteristics		Exp. (*n* = 26)	Cont. (*n* = 29)	z or *X*^2^ (*p*)
		**n (%)**	**n (%)**	
Sex^a^	Male	3 (11.5)	5 (17.2)	0.36 (.71)
	Female	23 (88.5)	24 (82.8)	
Age (years)		25.1 ± 8.1	23.5 ± 2.9	0.66 (.51)
Grade	3	13 (50.0)	15 (51.7)	0.02 (.90)
	4	13 (50.0)	14 (48.3)	
Part-time job experience^a^	Yes	24 (92.3)	28 (96.6)	0.48 (.60)
	No	2 (7.7)	1 (3.4)	
Allowance (Unit: 10 000 won)		53.54 ± 24.5	56.83 ± 29.0	0.49 (.62)
Participation in start-up education^a^	Yes	3 (11.5)	3 (10.3)	0.02 (1.0)
	No	23 (88.5)	26 (89.7)	
Participating institutions	University	2 (7.7)	2 (6.9)	-
	Others	1 (3.8)	1 (3.4)	

^a^Represents Fisher’s exact test results

### Effects of the 3S-BIS

**Fig. 2:**
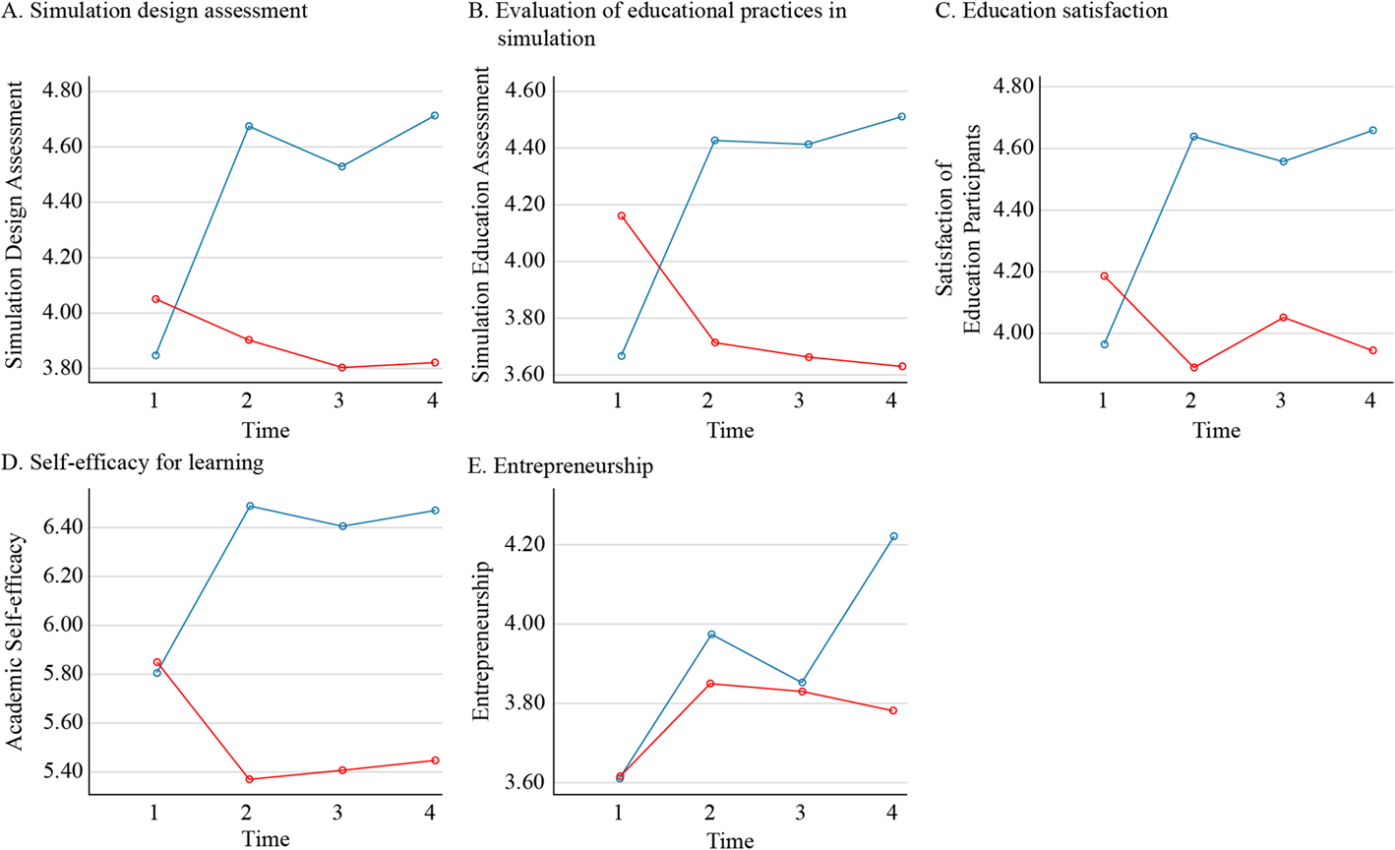
Graphs showing the differences in the effects of the program by time points

Hypothesis 1, “The experimental group that participates in the 3S-BIS program will have higher simulation design assessment (1–1), evaluation of educational practices in simulation (1–2), education satisfaction (1–3), self-efficacy for learning (1–4), and entrepreneurship (1–5) than the control group,” was supported. Compared to the control group, the experimental group had significantly higher simulation design score at T1 (immediately after intervention) and T3 (8 weeks after intervention), higher evaluation of educational practices in simulation at T1 and T3, higher education satisfaction at T1 and T3, higher self-efficacy for learning at T1 and T3, and higher entrepreneurship at T2 (Table 3 and Fig. 2).

**Table 3:** Effects of 3S-BIS with Friedman Test (*N* = 55)

Variable	Group	T0	T1	T2	T3	Friedman	T1–T0	T2–T1	T3–T2	T2-T0	T3–T0
		**M ± SD**	**M ± SD**	**M ± SD**	**M ± SD**	$\chi 2$**(*p*)**	**z (*p*)**	**z (*p*)**	**z (*p*)**	**z (*p*)**	**z (*p*)**
Simulation design assessment	Exp	3.85 ± 1.01	4.67 ± 0.39	4.53 ± 0.54	4.71 ± 0.35	30.60 (< .001)^*^	4.17 (< .001)	-1.66 (.096)	2.34 (.019)	-3.32 (.001)	4.20 (< .001)
	Cont	4.05 ± 0.65	3.90 ± 0.86	3.80 ± 0.95	3.82 ± 0.94	0.32 (.955)^*^	-1.09 (.274)	-0.46 (.648)	0.46 (.647)	1.11 (.269)	-0.59 (.559)
	z (*p*)	0.20 (.839)	-3.54 (< .001)	-2.80 (.005)	-3.88 (< .001)						
	Difference z (*p*)	**T1–T0**	**T2–T1**	**T3–T2**	**T3–T0**						
		-4.38 (< .001)	0.69 (.493)	-0.96 (.335)	-3.93 (< .001)						
Evaluation of educational practices in simulation	Exp	3.67 ± 1.12	4.43 ± 0.51	4.41 ± 0.48	4.71 ± 0.35	21.65 (< .001)^*^	3.84 (< .001)	-0.09 (.927)	1.06 (.291)	-3.37 (.001)	3.70 (< .001)
	Cont	4.16 ± 0.58	3.72 ± 0.89	3.66 ± 1.04	3.63 ± 1.08	8.00 (.046)^*^	-2.85 (.004)	0.09 (.925)	-0.15 (.879)	2.48 (.013)	-2.73 (.006)
	z (*p*)	1.49 (.137)	-3.06 (.002)	-2.69 (.007)	-3.34 (.001)						
	Difference z (*p*)	**T1–T0**	**T2–T1**	**T3–T2**	**T3–T0**						
		-4.79 (< .001)	0.08 (.933)	-0.86 (.388)	-4.66 (< .001)						
Education satisfaction	Exp	3.96 ± 1.04	4.64 ± 0.39	4.56 ± 0.44	4.66 ± 0.38	28.03 (. < 001)^*^	3.56 (< .001)	-0.86 (.388)	1.41 (.157)	-3.01 (.002)	3.69 (< .001)
	Cont	4.19 ± 0.57	3.89 ± 0.80	4.05 ± 0.74	3.94 ± 0.96	2.32 (.509)^*^	-2.05 (.041)	1.44 (.149)	-0.28 (.776)	1.08 (.279)	-1.05 (.296)
	z (*p*)	0.19 (.846)	-3.31 (.001)	-2.32 (.020)	-3.11 (.002)						
	Difference z (*p*)	**T1–T0**	**T2–T1**	**T3–T2**	**T3–T0**						
		-4.25 (< .001)	1.73 (.084)	-1.28 (.200)	-3.71 (< .001)						
Self-efficacy for learning	Exp	5.80 ± 1.26	6.49 ± 0.66	6.41 ± 0.78	6.47 ± 0.62	15.37 (.002)^*^	3.41 (.001)	0.21 (.836)	0.20 (.840)	-2.78 (.006)	3.51 (< .001)
	Cont	5.85 ± 0.77	5.37 ± 1.21	5.41 ± 1.24	5.45 ± 1.33	2.41 (.492)^*^	-2.20 (.028)	0.67 (.502)	0.42 (.676)	1.60 (.109)	-1.08 (.280)
	z (*p*)	-0.46 (.648)	-3.82 (< .001)	-3.17 (.002)	-3.04 (.002)						
	Difference z (*p*)	**T1–T0**	**T2–T1**	**T3–T2**	**T3–T0**						
		-4.01 (< .001)	0.81 (.420)	0.29 (.773)	-2.71 (.007)						
Entrepreneurship	Exp	3.61 ± 0.90	3.97 ± 0.73	3.85 ± 0.78	4.22 ± 0.76	12.09 (.007)^*^	2.51 (.012)	-0.55 (.586)	2.76 (.006)	-1.75 (.081)	3.02 (.003)
	Cont	3.62 ± 0.59	3.85 ± 0.81	3.83 ± 0.80	3.78 ± 0.83	5.87 (.118)^*^	1.64 (.101)	-0.14 (.893)	-1.62 (.105)	-1.65 (.098)	1.16 (.245)
	z (*p*)	-0.46 (.642)	-0.33 (.742)	-0.03 (.980)	-2.15 (.031)						
	Difference z (*p*)	**T1–T0**	**T2–T1**	**T3–T2**	**T3–T0**						
		-0.58 (.560)	0.40 (.691)	-3.04 (.002)	-1.93 (.053)						

^*^Significant differences with one another by Bonferroni correction *p* < .017

### Prolonged effects of 3S-BIS on start-up competency

Hypothesis 2, “Simulation design assessment (1–1), evaluation of educational practices in simulation (1–2), education satisfaction (1–3), self-efficacy for learning (1–4), and entrepreneurship (1–5) of the experimental group that participates in the 3S-BIS program will differ across time points,” was supported. Simulation design assessment score in the experimental group significantly differed between T1 (immediately after intervention) and T0 (baseline), and between T3 (8 weeks after intervention) and T0. Evaluation of educational practices in the simulation, education satisfaction, and self-efficacy also significantly differed between T1 and T0, and between T3 and T0. Entrepreneurship significantly differed between T3 and T2 (4 weeks after intervention), and between T3 and T0 (Table 3).

## Discussion

This section discusses the effects of the 3S-BIS program on various outcome measures among nursing students. To examine the effects of this program and the changes in the effects over time, we performed three post-tests after the program completion. The results showed that the experimental group had significantly improved simulation design assessment, evaluation of educational practices in simulation, education satisfaction, self-efficacy for learning, and entrepreneurship compared to the control group. Two participants were eliminated during the subject recruitment process because the program was not intended as education for technology start-up. Two participants were eliminated after the pre-test because of the overlap between department class hours and education hours. However, the elimination of the four participants was not regarded as having considerable impact on the results of this study.

Simulation design assessment and evaluation of educational practices in simulation evaluated by the experimental group, which participated in the 3S-BIS program, were significantly higher than those evaluated by the control group at T1 and T3. Currently, studies assessing simulation designs and educational aspects of simulation pertinent to start-up simulations are at an inchoate stage; thus, a direct comparison is difficult. Baek et al. reported that nursing students rated the design of a nasogastric tube management simulation as 4.33 and educational aspect of the simulation as 4.48 [17]. Han et al. employed a simulation education program to enhance forensic nursing competency and found that participants rated the simulation design as 4.42 points and simulation education as 4.78 points [23]. These are similar to scores of 4.67 and 4.43 for design and education, respectively, rated by the experimental group at T1 in the current study. These scores show that the simulation modules contain appropriate components and suggest that the quality of the simulation contents is crucial for start-up simulation education. Thus, start-up education should utilize a variety of teaching methods that include hands-on practices, as opposed to only using lectures that focus on imparting knowledge.

The experimental group showed a significantly higher education satisfaction and self-efficacy for learning than the control group at T1 and T3. Hung et al. provided simulation training involving emergency and critical care patients to nursing students and observed that students’ satisfaction and self-efficacy for learning significantly increased at six weeks after the simulation [24]. Saied also reported a positive correlation between simulation education and self-efficacy and education satisfaction [25]. These results suggest that the knowledge and experiences acquired from simulation training contribute to changing students’ behaviors and attitude. To boost nursing students’ self-efficacy and education satisfaction, various cases that emulate the actual cases in home-care nursing settings should be developed, and education should be provided with specific strategies that consider students’ current level.

The experimental group had significantly higher entrepreneurship at T3 compared to the control group. In a study on web-based start-up simulation education for nursing students, Lim et al. reported that entrepreneurship was significantly higher in the experimental group at T2 and T3 [15]. Entrepreneurship is an important determinant of the success of a start-up amid a rapidly evolving business environment, and start-up education can enhance individuals’ entrepreneurial attributes and foster an innovative start-up mindset [26]. A practical nursing start-up education that utilizes validated nursing start-up cases that impart practical information and tips for nursing start-up promotes successful nursing start-ups by increasing nurses’ entrepreneurship as well as their start-up competencies.

All of our study parameters—simulation design assessment, evaluation of educational practices in simulation, education satisfaction, self-efficacy for learning, and entrepreneurship—significantly improved immediately after the 4-week start-up nurses program intervention, and the improvement was retained until the 8-week follow-up. This result confirms that the effects of the 3S-BIS program are retained and that the simulation program is an appropriate practical training and educational tool that boosts nursing students’ nursing start-up competencies.

Simulation design assessment, evaluation of educational practices in simulation, education satisfaction, self-efficacy for learning, and entrepreneurship are inter-related. With start-up education being provided more frequently, there is a growing number of systematic studies on the development and designing of simulation curricula as well as studies investigating the effects of education [27]. Systematically designed simulation training enhances self-efficacy for learning, a belief on one’s ability to utilize newly learned contents [19], which increases students’ overall satisfaction. Facilitating learning through active interactions with students, as opposed to simply adopting the role of delivering knowledge, increases students’ satisfaction with learning [28]. Improved entrepreneurship as a result of learning serves as the psychological foundation that drives nursing start-ups. The findings of this study show that the 3S-BIS program is empirically effective in cultivating nursing start-up competency in nursing students.

The 3S-BIS program was designed to utilize simulation training to overcome the limitations of lecture-based learning. One key benefit of simulation is that students can practice a topic that otherwise cannot be practiced in an actual life setting in a safe and standardized environment, and that they can repeatedly practice it until they master it [29]. In recent years, simulation has been increasingly utilized in practicum courses in various aspects of nursing curricula in South Korea and abroad [17, 28]. In particular, hands-on education and training are essential for start-up education unlike other subjects that can be taught through methods that focus on imparting knowledge, further highlighting the importance of simulation in start-up education [27].

With the increased use of online learning because of the COVID-19 pandemic, there have been voices raising concerns about learning outcomes and instructor-learner interactions, and discussions about effective and efficient teaching and learning methods are ongoing. Moreover, a nursing curriculum includes both theory and practicum courses for third- and fourth-year students; thus, more student-friendly approaches are needed when providing nursing start-up education to third- and fourth-year students. The 3S-BIS involves a web-based simulation training that allows students to acquire knowledge about nursing start-ups and develop critical thinking and problem-solving skills through simulation training. Students generate results based on the simulation scenarios and are debriefed by their instructor, which helps them interact with their instructors and review their learning outcomes. A web-based self-study module that allows students to learn at their own pace without restrictions of time and space probably contributed to increasing the learning effects. These components of the program helped nursing students to be equipped with all knowledge and experience needed to start their own business, thereby fostering a positive attitude and confidence in nursing start-ups. However, start-up competency is not something that can be altered in a short period; hence, a mentoring system in which students are continually remotivated and supported should be incorporated to start-up education programs.

Kim stated that support for nursing start-ups is essential such that positive nursing experience, nursing competencies, and nursing start-up performance can lead to nurses’ starting a business [30]. Thus, nursing schools should continuously expose students to nursing start-ups through training and site tours and continue to attempt various experimental processes, such as development and implementation of diverse nursing start-up simulation curricula. Furthermore, scenarios that engage students and start-up learning activities and teaching strategies that allow students to collaborate with their colleagues should be developed.

Finally, since most participants in the current study are women, follow-up studies to confirm the difference in educational effects according to sex are needed in the future. Although the effect of 3S-BIS education was confirmed through repeated measurement, future studies should confirm the timing of booster education for the continuation of the educational effects. This study measured the effect of nursing start-up education using 3S-BIS on nursing students; however, since 3S-BIS was developed as an ERP, future research to measure the management effect of visiting nursing centers is needed.

## Conclusions

The 3S-BIS program was designed to offer practical support for nursing students to start a nursing business and cultivate nursing leaders. The program was designed to teach about the basic structure of running a home healthcare service, and it accounted for the fact that the services are provided at client’s homes and that nurses are not management experts. Thus, the significance of this study is in structuring the program and developing an operating system to realize the three Ss—Smart, Speed, Simple. Furthermore, we used a sophisticated study design—a repeated-measures RCT—to analyze the effects of the 3S-BIS program on nursing students’ rating of simulation design and educational aspect of stimulation, education satisfaction, self-efficacy for learning, and entrepreneurship. The program can be utilized in various formats for nursing start-up education as part of the nursing curriculum, and we confirmed that the program is appropriate for achieving the learning objectives and resolving the anticipated problems that are encountered by home healthcare services.

This study had some limitations. The study subjects were limited to Korean nursing students, so caution needs to be exercised in generalizing the study results. Considering that the intervention period in this study was only one week, follow-up research comparing various study periods should be conducted in the future. In addition, the study does not discuss the effectiveness of various entrepreneurship competency enhancement programs such as entrepreneurship theory education courses, practical entrepreneurship courses, and entrepreneurship mentoring. Thus, we suggest that future studies should verify the effectiveness of these programs. Prospective cohort studies are needed to determine whether nursing students who complete the 3S-BIS program actually start a business, and the study sample should be expanded to nurses who operate or work in a home health care service to verify the effects of the program on these nurses.

## Data Availability

The data generated in this study are openly available in Mendeley Data at https://data.mendeley.com/datasets/v3ffh3c399, reference number DOI-10.17632/v3ffh3c399.1.

## Abbreviations

BIS: Business intelligence system
ERP: Enterprise resource planning
KRW: Korean won
LTC: Long-term care
RCT: Randomized controlled trial
USD: United States dollar
